# Assessment of Disruption of Routine Childhood Immunization in Developing Countries Due to Pandemic

**DOI:** 10.7759/cureus.30845

**Published:** 2022-10-29

**Authors:** Riddhi S Poshattiwar, Ashish Anjankar

**Affiliations:** 1 Community Medicine, Jawaharlal Nehru Medical College, Datta Meghe Institute of Medical Sciences, Wardha, IND; 2 Biochemistry, Jawaharlal Nehru Medical College, Datta Meghe Institute of Medical Sciences, Wardha, IND

**Keywords:** covid19 pandemic, developing countries, vaccine-preventable diseases, routine immunization unimmunized children, disruption

## Abstract

The COVID-19 pandemic, also known as the coronavirus pandemic, began in March 2020 and was caused by severe acute respiratory syndrome coronavirus 2 (SARS-CoV-2). The pandemic impacted the global healthcare system. It caused the biggest threat to the global routine immunization system. Routine childhood immunization was disrupted globally, particularly in the early pandemic period. This review discusses the severity of disruptions to routine immunization, their root causes, and remedial measures to lessen these disruptions. It is essential to maintain routine medical care, especially routine immunization, to avert morbidity and death from several diseases that vaccines can prevent, including a pandemic. The healthcare system's reaction to a pandemic must include catch-up vaccinations because missed vaccines increase the population's and children's health risks.

## Introduction and background

Severe acute respiratory syndrome coronavirus-2 (SARS-CoV-2) caused the COVID-19 pandemic also called coronavirus pandemic [1]. The novel coronavirus's first case was reported in China's capital, Wuhan, in December 2019 [2]. On January 30, 2020, the World Health Organization announced a Public Health Emergency of International Concern [3]. The coronavirus pandemic was announced on March 2020 by the World Health Organization [4]. In January 2020, India reported its first case. On March 12, 2020, the Indian Government announced its first fatality from COVID-19 [5]. The honorable Prime Minister of India announced a one-day curfew on March 22, 2020. India entered a state of emergency on March 25, 2020, lasting through April 14 [6]. With the request for lockdown, the healthcare systems began to prioritize COVID-19 care over other healthcare needs. The immunization program is one of the most crucial methods for preventing diseases that can kill children yet are avoidable. It is a significant public health endeavor for the country and one of the most incredible vaccination schemes in the world. The Expanded Immunization Program was the name of India's vaccination program in 1978. In 1985, the Expanded Immunization Program was changed to the Universal Immunization Program [7]. Globally due to the pandemic caused by coronavirus, the Universal Immunization Program was at risk of disruption due to the restrictions and isolation steps established to attenuate the pandemic caused by coronavirus [8]. The United Nations International Children's Emergency Fund, the Vaccine Alliance, the Global Alliance for Vaccines and Immunization, and the World Health Organization have announced that routine immunization programs have been badly disarranged in almost 68 countries, influencing about 80 million children [9]. More than half of the 129 nations for which statistics were available announced mild to severe disarrangements, or a complete end, of immunization facilities from March to April 2020 [9].

## Review

The pandemic impacted global economics, politics, culture, education, the environment, and the global healthcare system [10]. Due to the isolation and restriction measures implemented to contain the coronavirus pandemic, the Universal Immunization Program was at risk of being disrupted. The National Immunization Program includes bacillus Calmette-Guerin vaccine (BCG), oral polio vaccine (OPV), pentavalent vaccine, rotavirus vaccine, pneumococcal conjugate vaccine (PCV), fractional inactivated poliomyelitis vaccine (fIPV), measles-rubella vaccine (MR), and Japanese encephalitis vaccine (JE). The pentavalent vaccine protects from diphtheria, pertussis, tetanus, hepatitis B, and Haemophilus influenza type B. The National Immunization Program is in charge of reducing under-five vaccine-preventable mortality. It is notably the most important low-cost public health intervention. If a child receives all recommended vaccinations under the National Immunization Program before age one, the child is considered completely immunized [11]. In June 2020, 83% of the 424 doctors claimed that immunization services decreased by 50% [12]. As stated by one study, 76% of pediatricians voiced concern about the routine immunization coverage gap [12]. According to a study conducted in 19 countries in the South-East Asia and Western Pacific region, routine childhood immunization programs were disrupted in 95% of these countries [13]. According to official data released by the World Health Organization (WHO) and the United Nations Children’s Fund (UNICEF), 23 million children (3.7 million more than in 2019) failed to receive essential immunizations through routine immunization services in 2020 [14]. The number of children in all regions who did not receive their first immunizations rose as access to health care and immunization outreach were restricted [14]. Most children who did not receive their routine immunizations reside in underserved distant locations, conflict-affected communities, slum areas, or other informal settings, where they must deal with various challenges, such as limited access to essential social and medical services [14].

According to a report that collected data from 170 countries and territories, the month of April 2020 saw the fewest vaccination doses given globally, with a 33% decrease in the diphtheria-pertussis-tetanus vaccine (DTP3), with decreases ranging from 9% in the World Health Organization African region to 57% in the South-East Asia region [15]. The first dose of the measles-containing vaccine (MCV1) and diphtheria-tetanus-pertussis vaccine (DTP3) provided in the first half of 2020 was mentioned in a report that compiled data from various countries and territories. Globally, the estimated vaccine coverage for DTP3 and MCV1 in 2020 was 76.7% (95% confidence interval: 74.3%-78.6%) and 78.9% (74.8%-81.9%), respectively. These percentages represent 7.7% (6.0%-10.1%) for the DTP3 vaccine and 7.9% (5.2%-11.7%) for the MCV1, differentiated to anticipated doses administered when the COVID-19 pandemic was not present [16]. By June 2020, vaccine recovery had started and lasted until the end of 2020 [15]. Here is an overview of how the pandemic has affected routine childhood immunization in developing countries.

Impact in Nepal

In Nepal, almost 50% of immunization centers closed facilities during the inception of the lockdown [17]. Services were abruptly stopped in March when patients began to appear among healthcare professionals [17]. A study in Nepal revealed that families moving from urban to rural areas during the pandemic made it difficult for facility suppliers to confirm the immunization status of migratory children. Additionally, it has been hypothesized that the lockdown measures may negatively affect Nepal's decade-long significant gains in child health, mainly the routine childhood immunization services [18]. To lessen the impact of disrupted routine immunization, the Ministry of Health and Population of Nepal instructed its Family Welfare Division to put safety precautions in place and adjust service delivery as needed to continue routine immunization services throughout the nation's prolonged lockdown (i.e., end of March through most of July 2020) [17].

Impact in Africa

In Africa, an intensive analysis of the chances of the eruption of disease caused by morbillivirus during the pandemic was assessed in Kenya. Population immunity was almost near the herd immunity threshold in February 2020, when a scheduled supplementary immunization activity was postponed, and the likelihood of a large outbreak was 34% (8%-54%) [19]. The probability of a significant measles outbreak will rise to 38% (19%-54%), 46% (30%-59%), and 54% (43%-64%) from December 2020 as the COVID-19 contact restrictions are almost completely relaxed, assuming 15%, 50%, and 100% decline in measles vaccination coverage [19]. The conclusion was that organizing a supplementary immunization activity with a 95% scope in children under five can reduce the probability of a measles outbreak if all restrictions are lifted [19]. Postponing the scheduled drives in Ethiopia and Nigeria by one year (both countries completed their supplementary immunization actions planned for 2020) could significantly raise the risk of measles outbreaks [20]. If yellow fever vaccination programs are put off, the disease burden could rise by more than one death per 100,000 people every year up till the drives are put into place [20].

Impact in Bangladesh

Bangladesh's immunization was also impacted due to the pandemic. A study done in Bangladesh found that April and May 2020 experienced the worst setbacks to child immunization programs, with 20% (280 of 1,414) and 25% (346 of 1,395) of the scheduled outreach vaccination facilities being called off, respectively [21]. In order to prevent further disruption of routine childhood immunization, the district health management and local ministry of health authorities should train service providers and utilize resources from other programs [21].

Impact in India

A study conducted in Rajasthan, India, reflected that children during the lockdown had considerably lower odds of receiving a vaccination at or before the age of nine than children before the lockdown. Still, they had higher odds of receiving one between 10 and 12 months [22]. Additionally, they were unlikely to have had all of their essential first-year vaccinations by the time of the study [22]. First-year vaccination coverage fell from 9.7% to 14.0% in children during the lockdown [22]. In India, a study was conducted in Meerut, Uttar Pradesh, to analyze the impact of the pandemic on immunization. They discovered that compared to February 2020, the total population receiving routine immunization in August and December 2020 had experienced a drop [23]. The doses given at birth were the least affected. Vaccine coverage of birth-dose bacillus Calmette-Guerin (BCG), oral polio vaccine (OPV), and hepatitis B declined by 27.9%. Coverage of the first dose of the measles-rubella vaccine (MR1) decreased by 68.42%, and the second dose of the measles-rubella vaccine (MR2) declined by 84.3%. A decline of 57.4% was seen in the vaccine coverage of the second booster dose of the diphtheria-pertussis-tetanus vaccine (DPT b2) [23]. Important vaccination-related characteristics, like community accessibility, facility readiness, and immunization intention, were impacted by the COVID-19 lockdown. Causes of disruption of routine childhood immunization are given in the following sections.

Healthcare Workers Related

**Figure 1:**
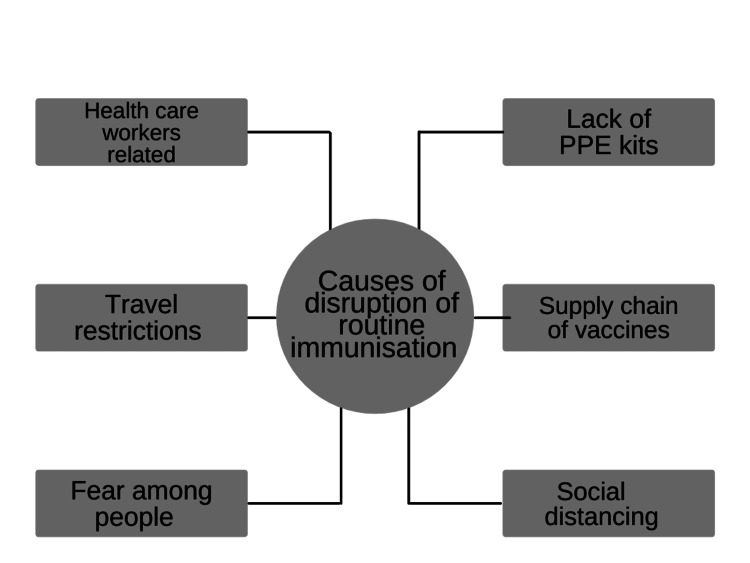
Causes of disruption of routine childhood immunization The author has recreated from the sources [23-25]. PPE: personal protective equipment.

Healthcare workers have tested positive for the virus or died due to the COVID infection. Moreover, public transport was also unavailable for healthcare workers to travel from home to work [23] (Figure 1).

Insufficient Number of Personal Protective Equipment Kits

In March and April 2020, there was a lack of personal protective equipment kits, so India needed to act quickly to manufacture and buy crucial personal protective equipment [23] (Figure 1).

Travel Restrictions for the General Population

During the pandemic, public transportation was unavailable and unsafe to use without masks. The parents were discouraged from taking their kids to routine immunization [23] (Figure 1).

Vaccine Type

The problem with multi-dose vials is that healthcare professionals decide not to open them when few recipients show up for vaccinations. The above issue is especially true for vaccines such as the measles-rubella vaccine and Japanese encephalitis vaccine that do not have an open-vial policy [23] (Figure 1).

The Supply Chain of Vaccines

Pressure is being put on worldwide manufacturer production capabilities, supply availability, and logistics as a result of the current coronavirus outbreak. Additionally, it jeopardizes the continuation of vaccination campaigns in nations due to lockdowns and other precautions taken to stop the virus's spread, which also affects supply [24].

Fear Among People

People are not usually reluctant to get immunizations for several reasons, but resistance has recently raised significant concerns. Parents were worried about contracting the disease while visiting a hospital for routine immunization after the outbreak [25] (Figure 1).

Social Distancing Guidelines

The guidelines from the government on physical distancing made it very difficult to perform established hospital-based activities like routine immunization [23] (Figure 1).

Containment measures for the virus spread included sudden and unscheduled cancellations of schools during the lockdown [26]. The sudden closure of schools is concerning since these locations are crucial for providing young children with various health treatments, including vaccinations. Due to the lockdown, all the workers in big cities migrated back to their hometowns for basic survival [27]. The workers moved back to their hometowns along with their families. Children from these families missed out on the vaccination. The World Health Organization's Strategic Advisory Group of Experts on immunization recommended delaying further preventive mass inoculation campaigns for other diseases during the pandemic because of social exclusion concerns [28]. It is essential to observe the cases and the deaths of the patients caused by other than the pandemic as these other cases are a significant burden for healthcare [29]. Proof from the epidemic caused by Ebola (2014-2015) also reflects that more deaths from infections other than Ebola were attributed to a flawed healthcare system [30]. Even during an epidemic that has crossed international boundaries, it is critical to maintain routine health services, including routine immunization, to prevent morbidity and death from other diseases which can be prevented by vaccination [31]. Due to the time-sensitive nature of childhood vaccinations, even a minor delay in the immunization status of millions of children will jeopardize the results of decades of hard work [24]. During and after a pandemic, the healthcare system's response must include catch-up immunization because missed vaccination raises the population's and children's health risks [32,33]. However, they significantly increase the demands on already few resources [34,35]. The aperture in routine childhood immunization needs to be corrected by taking prompt steps to immunize due recipients, which will help reverse the interference or hindrance of vaccination on the future of India's immunization program [36]. The COVID-19 pandemic has demonstrated how crucial it is for responses and uptake to have precise and timely information about resource allocation. Similar circumstances apply to routine immunization services, where information routinely gathered on children who missed their vaccination can guide both individual and group catch-up efforts [16]. The pandemic crisis stopped the Indian Government's Intensified Mission Indradhanush 2.0 from attaining its aim of vaccine coverage [37]. The Indian Ministry of Health and Family Welfare published instructions on routine immunization services during the pandemic. According to the recommendations, birth dosages should continue in containment and buffer zones, while routine immunization sessions and outreach activities should be discontinued. Birth dosages, routine immunization sessions, and outreach activities should continue in regions outside the buffer zone. Measures that need to be followed for successful immunization services during the pandemic are as follows: a distinct area of demarcation for incoming beneficiaries, a waiting area after vaccination, a reserve zone in case gathering rises, and pre-identification of session site with enough seats for beneficiaries and caretakers while keeping a social distance of at least 1 m [38] (Table 1).

**Table 1:** Immunizations processes continued or discontinued in different zones The author has recreated from the source [38].

	Containment zone	Buffer zone	Beyond buffer zone	Green zone
Birth dose	Continued	Continued	Continued	Continued
Health facility-based session	Discontinued	Discontinued	Continued	Continued
Outreach session	Discontinued	Discontinued	Continued	Continued

To build on the achievements of essential vaccination to cover any vaccination aperture that may have been created due to the increased number of coronavirus cases, Intensified Mission Indradhanush 3.0 was started by the Indian Government [39]. The following recommendations are made to prevent the disruption of routine childhood immunizations: real-time data should be used to identify children who missed their immunizations, surveillance of diseases that can be prevented by vaccination should be strengthened and maintained, routine immunization services should be provided at home or at drive-through locations, the importance of routine childhood immunization and the risks associated with it should be promoted when the vaccinations are missed, communication abilities of those administering the vaccines must be strengthened by using this pandemic as a teaching tool, invention of digital tools to track the people who migrated during the pandemic, and collaboration and capacity building between non-government organizations and the private health sector will help to accomplish the goal [23].

## Conclusions

World Health Organization declared a coronavirus pandemic in March 2020. The pandemic's most significant effect was on routine childhood vaccination. Globally, the routine immunization program was disrupted. Causes of disruption of immunization include supply side issues, lack of personal protection equipment kits, travel restrictions for the general population, type of vaccine, supply chain of vaccines, fear among people, and social distancing guidelines. We must ensure that routine immunization delivery is given enough resources and attention, while governments, healthcare professionals, and academics are rightfully focusing on the immediate response to the COVID-19 pandemic. During and after a pandemic, the healthcare system's response must include a catch-up vaccination program because missed vaccinations raise the population's and children's health risks. In India, to catch up with the vaccination pace, the Indian Ministry of Health and Family Welfare published instructions on services for routine immunization during and after the pandemic. Real-time data and careful tracking are necessary to ensure children have completed their routine immunization. Some other recommendations to prevent further disruption of routine childhood immunization include promoting the importance of routine childhood immunization and the risks associated when the vaccinations are missed, drive-through clinics or home visits for providing the immunization services, improving the communication skills of the people who administer the vaccines, and collaborations between non-government organizations and the private health sector. An emergency preparedness plan may be created to prevent such a disruption to the routine immunization program in the event of a future pandemic or calamity.
